# Estimation of vegetation indices for high-throughput phenotyping of wheat using aerial imaging

**DOI:** 10.1186/s13007-018-0287-6

**Published:** 2018-03-14

**Authors:** Zohaib Khan, Vahid Rahimi-Eichi, Stephan Haefele, Trevor Garnett, Stanley J. Miklavcic

**Affiliations:** 1 Phenomics and Bioinformatics Research Center, University of South Australia, Mawson Lakes Boulevard, Adelaide, 5095 Australia; 2 School of Agriculture, Food and Wine, University of Adelaide, Adelaide, 5064 Australia

**Keywords:** Wheat, Phenotyping, Deep learning, Precision agriculture

## Abstract

**Background:**

Unmanned aerial vehicles offer the opportunity for precision agriculture to efficiently monitor agricultural land. A vegetation index (VI) derived from an aerially observed multispectral image (MSI) can quantify crop health, moisture and nutrient content. However, due to the high cost of multispectral sensors, alternate, low-cost solutions have lately received great interest. We present a novel method for model-based estimation of a VI using RGB color images. The non-linear spatio-spectral relationship between the RGB image of vegetation and the index computed by its corresponding MSI is learned through deep neural networks. The learned models can be used to estimate VI of a crop segment.

**Results:**

Analysis of images obtained in wheat breeding trials show that the aerially observed VI was highly correlated with ground-measured VI. In addition, VI estimates based on RGB images were highly correlated with VI deduced from MSIs. Spatial, spectral and temporal information of images contributed to estimation of VI. Both intra-variety and inter-variety differences were preserved by estimated VI. However, VI estimates were reliable until just before significant appearance of senescence.

**Conclusion:**

The proposed approach validates that it is reasonable to accurately estimate VI using deep neural networks. The results prove that RGB images contain sufficient information for VI estimation. It demonstrates that low-cost VI measurement is possible with standard RGB cameras.

## Background

Satellite multispectral imaging has demonstrated the ability to efficiently map Earth’s resources (vegetation, water, minerals etc.) from remote locations [1, 2]. Recent technological advances in imaging methods are moving agricultural practice from traditional farming to precision farming. The unmanned aerial vehicle (UAV) platform is becoming an important tool for field-based precision agriculture [3–5]. Lightweight, high-resolution imaging sensors have been developed and can be used with most UAVs [6, 7]. Aerial platforms can be used to support computerized ground-based vehicles in the management of extensive agricultural lands. Precise spatial application maps can then be developed to direct ground based remedial measures to increase production efficiency. The result is a site specific agricultural management solution based on aerial observations.

A UAV equipped with a multispectral camera can be used to monitor spatial and temporal variations in vegetation characteristics. A vegetation index (VI) is a spectral transformation metric for measuring the presence and state of vegetation [8]. Its basis is the characteristic photosynthetic response of green vegetation to incident light. Healthy plants exhibit high infrared reflectance and low red reflectance due to absorption of red light by chlorophyll, resulting in a high index value. Conversely, unhealthy, stressed or dead vegetation, a manifestation of reduced chlorophyll pigment, displays a low index value. Therefore, VI measures can be used to facilitate corrective measures in crop management.

Various uses of VI for the detection of biotic and abiotic stresses have been demonstrated. Vegetation indices were correlated with soil moisture measurements to assess the sensitivity of tallgrass prairie grasslands to drought [9]. The indices allowed remote identification of drought affected regions and could potentially be used to quantify the effects of drought on vegetation. Vegetation indices also have the potential to differentiate healthy from diseased plants [10]. Targeted application of insecticides and herbicides which is of immense value to agricultural economics can be automatically carried out by a UAV capable of both observation and treatment application. Apart from stress, VI were shown to be sensitive to phenological changes (e.g. senescence) with age [11]. As a result it was possible to predict the age of plant leaves in forest to assess the state of ecosystem. Gracia-Romero et al. [12] evaluated several aerially assessed and ground based VI and found them to be highly correlated with Maize performance with fertilization. In addition, vegetation indices have demonstrated correlation with several performance characteristics of crops including biomass, yield potential and nutrient concentration [13, 14].

Vegetation indices are generally computed as the ratio of difference to sum of the sensor measurements in two bands. One of the most widely known is the Normalized Difference Vegetation Index (NDVI) [15], extensively used since the introduction of LANDSAT-1 satellite multispectral data. NDVI is based on measurement in Red and Near Infrared (NIR) channels to identify regions of vegetation cover and their condition. Other empirically derived indices based on the same principle make use of different bands in the photosynthetically active spectral range in combination with NIR [16]. The main idea is to maximize sensitivity to vegetation and minimize the noise. The RedEdge Normalized Difference Vegetation Index (RENDVI) is most sensitive to leaf area and less prone to index saturation [17]. The Soil Adjusted Vegetation Index (SAVI) aims to minimize the influence of soil reflectance in computation of VI by adding a background adjustment factor [18]. The Enhanced Vegetation Index (EVI) further corrects for atmospheric noise by introducing aerosol resistance factors [19]. Although important for remote satellites, atmospheric noise is an insignificant factor for UAV imaging. Table 1 lists the common multispectral VIs found in literature.

**Table 1:** A list of commonly used VIs in literature

Vegetation index	Formula
Normalized Difference Vegetation Index [15]	${\text{NDVI}} = \frac{{{\text{NIR}} - {\text{Red}}}}{{{\text{NIR}} + {\text{Red}}}}$
Green Normalized Difference Vegetation Index [16]	${\text{GNDVI}} = \frac{{{\text{NIR}} - {\text{Green}}}}{{{\text{NIR}} + {\text{Green}}}}$
RedEdge Normalized Difference Vegetation Index [17]	${\text{RENDVI}} = \frac{{{\text{NIR}} - {\text{RedEdge}}}}{{{\text{NIR}} + {\text{RedEdge}}}}$
Soil Adjusted Vegetation Index [18]	${\text{SAVI}} = \frac{{(1 + {\text{L}}) \times ({\text{NIR}} - {\text{Red)}}}}{{{\text{NIR}} + {\text{Red}} + {\text{L}}}}$
Enhanced Vegetation Index [19]	${\text{EVI}} = \frac{{{\text{G}} \times ({\text{NIR}} - {\text{Red}})}}{{{\text{NIR}} + {\text{c}}_{1} {\text{Red}} - {\text{c}}_{2} {\text{Blue}} + {\text{L}}}}$

It is clear from the above definitions that the NIR reflectance is a critical requirement, common to most VI. However, the NIR channel is not available in standard RGB cameras. UAVs equipped with RGB cameras are therefore incapable of providing a direct VI measure. A straightforward solution is a 4-channel camera with the additional NIR channel, usually known as a multispectral camera. However, multispectral cameras compatible with UAVs come with a very limited spatial resolution (< 5 million pixels), compared to most RGB cameras (up to 20 million pixels). Although high resolution may not be crucial for accurate NDVI measurement, it is desirable for many image phenotyping tasks such as flower detection, plant height [20] and leaf coverage estimation. Multispectral cameras have relatively low spatial resolution for such tasks. This compels the end-user to either tradeoff spatial resolution for spectral resolution, or conduct multiple flights with each sensor separately, to achieve both targets. Some UAVs allow for simultaneously carrying multiple sensors (owing to payload limitations). Such a system would require accurate synchronization, alignment and integration of the sensors. This is a challenging task in dynamic scenarios where vegetation movement is inevitable due to environmental factors.

To circumvent costs, the NIR filter present inside a standard RGB camera can be removed. The implication of such a modification is a camera with blue, green and NIR channels. Then, the tradeoff is to use the blue channel to simulate the absorption in red channel. However, the blue channel in most camera sensors is prone to low signal to noise ratio. In addition, the equivalence of absorption in two different channels may not be necessarily true. An improvement over this design is to remove the NIR filter from an RGB camera and introduce an additional high-pass filter, which theoretically results in NIR, green and red channels [21]. The optimal filter parameters for a specific camera are set to minimize the difference between reference and target spectral values. However, this requires careful customization of camera and measurement of the camera sensitivity function. Yet another approach is to remove the NIR filter and introduce a dual band-pass filter to enhance NDVI measurement [22]. A major drawback as a consequence of modification of an RGB camera is the unavailability of an original RGB image. Apart from retrofit modifications, commercial dual CCD sensors, each with a different color filter to target desired channels have been compared with single CCD sensor with multiple color filters for VI measurement [23]. However, the modifications add to the design and production costs limiting their use to specialized applications.

Although a number of methods for the modification of camera hardware have been proposed, each with its own benefits, this paper proposes a model-based approach to estimating VI from RGB images. The main idea is to learn the spatio-spectral relationships between information in RGB images of vegetation and their corresponding VI values (sourced from MSI). This is achieved by leveraging a deep neural network (DNN) to model the non-linear relationship between an RGB image and its vegetation index. Deep learning is classified as a machine learning method for learning multi-level representations of data [24]. It has performed well on a wide range of plant phenotyping tasks like organ counting [25–27], age estimation [28], feature detection [29, 30], species and disease detection [31, 32]. Our motivation to use DNN was to formulate a regression problem such that the multilayered convolutional features learned by the model relate RGB image data to NDVI. The rationale of our proposed approach is simple but effective, i.e. the spatial density and spectral signature (color) of vegetation reflects its VI.

Limmer and Lensch investigated a contrasting problem of colorizing infrared images [33]. They used DNN to synthesize RGB image of a scene from its infrared counterpart with reasonable visual quality. Our approach to estimating VI is distantly similar to the band simulation approach proposed by Rabetal et al. [21]. The major difference that distinguishes our work is that it does not rely on camera hardware modification and extensive camera sensitivity measurements. Moreover, the use of an unmodified camera allows for retaining the high-resolution RGB image for useful purposes while simultaneously achieving a VI estimate. There are no additional costs associated for the purpose of such application. To the best of our knowledge, this is the first attempt on modeling vegetational indices from color images using deep learning.

## Methods

### Breeding experiment

The trial site used for this study was in a farmers’ field between Mallala and Balaklava, South Australia (Lat:34°18′4.29012″:S Long:138°28′57.05255″:E). A wheat breeding trial was conducted on site in collaboration with a wheat breeding company (LongReach Plant Breeders). The experiment was sown on 25th May, 2016, and harvested at 214 days after sowing (DaS). A total of 1728 single row wheat plots on the site were considered at 7 growth stages, resulting in 12096 observations.

**Fig. 1:**
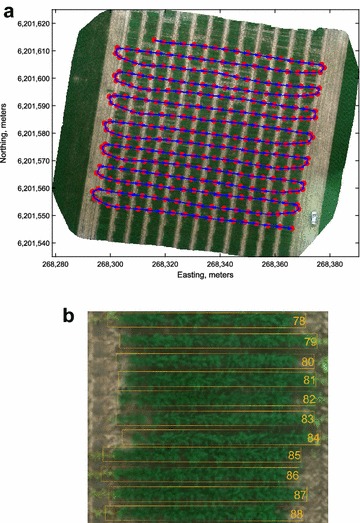
Experimental site. **a** UAV imagery of the trial site mapped to UTM Zone 54S in WGS84 coordinate system. Blue arrows indicate the flight direction and red markers indicate imaging points. **b** Rectangular grid aligned to single row plots and displayed as an overlay

The three ‘bays’ monitored on the site were part of a much larger trial which was almost 1400 m long and 72 m wide. Each bay was 19.2 m long and had 12 ranges (12 × 6 m) Germplasm entries were planted in double rows, perpendicular to the ranges, with a row spacing of 0.4 m, such that 24 entries were planted in each range making a total of 288 entries in a bay (see Fig. 1a). The initial length of planted rows was 5 m which was later sprayed back to 4 m. Therefore, each individual entry had two rows of 4 m and an area of 3.2 m^2^. It was noted after emergence that some rows were shorter due to insufficient seed at the time of sowing.

The first bay (coded R40) was planted with double haploid (DH) lines of an EGA Gregory/Spitfire population targeted at studying the genetic control of grain N concentration. In this experiment, the DH entries were replicated twice and a total of 94 DH lines were tested. Along with the DH lines, 19 soft wheat check varieties, 02 hard wheat check varieties, and both hard wheat parents were planted in a fully randomized layout. The remaining plots in this bay were filled with germplasm not related to the trial. The next two bays (coded R41 and R42) were planted with DH lines of 6 different crosses of soft wheat (3 in each bay), which were part of the soft wheat breeding program. For each cross, 80 different DH lines were grown unreplicated, together with 24 twice replicated check varieties of soft and hard wheat. In addition, a highly disease susceptible line (Morocco) was regularly repeated in each range to increase the disease pressure. All DH lines of each cross were grown in a block (4 ranges within a bay) and check varieties were randomized within this setup.

### Ground reference

Ground based NDVI was estimated using a handheld crop sensor, ‘GreenSeeker’ (Trimble, USA). The measurements were conducted by making a continuous sweep from the start to the end of a plot. A constant height and position over the center of an entry (i.e. the middle of two rows) was ensured by adjusting a thin line with a small weight on the sensor. Two lines in each bay were selected, and the 12 plots behind them were measured, so a total of 24 plots were measured in each bay. The measurements were conducted on the following DaS: 93, 117, 141, 156, 170, and 182.

### UAV image acquisition

A 3DR Solo (3D Robotics Inc., USA) drone was used with a custom platform to attach a RedEdge™MultiSpectral camera (MicaSense Inc., USA). The camera was capable of simultaneously capturing five spectral bands at a resolution of 1.2 megapixels. For flight planning and automatic mission control an open source autopilot software Mission Planner (ArduPilot) was utilized. The multispectral camera was set to auto-capture mode with one image every two seconds. Image overlap was always ≥ 80% at a constant speed of ≤ 3 ms^−1^, but the actual speed could vary depending on the selected flight altitude, image capture rate and requested overlap. Initially, images were only acquired from 30 m height, but from the fifth session onwards, images were also taken from a lower 20 m height for increased ground resolution.

A total of seven imaging sessions were conducted at intervals ranging from 1 to 3 weeks between August and November of 2016. A trial imaging session was conducted on DaS: 72, and regular imaging sessions were planned thereafter. However, the actual dates were adjusted according to suitable weather conditions (bright and not too windy). Subsequent imaging sessions were conducted at DaS: 93, 113, 135, 141, 156, 170, and 182.

In order to geographically register images captured in multiple sessions, 12 square panels were placed at fixed positions in the surveying area to serve as ground control points (GCP). The GCPs were repeatedly placed at the same position before commencement of an imaging session, throughout the season. An image of a calibrated reflectance panel (MicaSense Inc., USA) was also captured from directly overhead the panel before and after each flight for radiometric calibration. All raw images were stored in a 16-bit TIFF file format.

### UAV image processing

The acquired images were imported into Pix4D mapper v3.2 (Pix4D Inc., Switzerland) for offline processing. Camera correction and calibration was applied to remove geometric distortions from images. Finally, a stitched orthomosaic image was generated with a Ground Sampling Distance (GSD) ranging between 2.0 cm (30 m altitude) to 1.3 cm (20 m altitude). The orthomosaic image was radiometrically calibrated with the image of the standard white reflectance panel. Coordinates of the GCPs were used to compute geometric image transformation required to geographically register orthomosaics of successive imaging sessions. The calibrated orthomosaics were imported into MATLAB R2017a (Mathworks Inc., USA) for sampling of the reflectance data of plots. A uniform rectangular grid of fixed dimensions was laid out and aligned with the ground plot locations (see Fig. 1b). The geographic coordinates of rectangles were converted to intrinsic image coordinates to automatically crop the individual plot images. A few sampled plots were missing image data due to being outside the mapped range of UAV on DaS 135. These 18 images were excluded from the analysis. Since the orthomosaic images of different dates varied in resolution, the sampled images were scaled to a uniform size of 208 × 15 pixels.

**Fig. 2:**
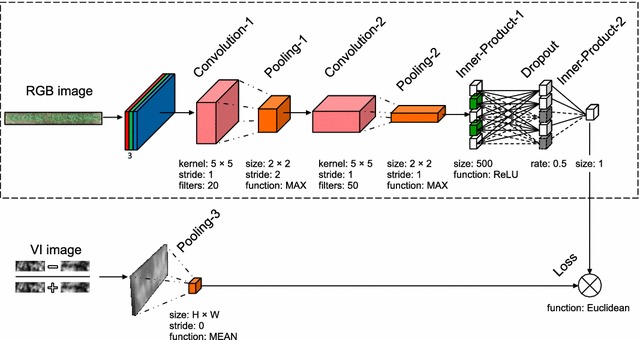
Deep neural network architecture. A schematic illustration of the deep neural network for index estimation. Dotted rectangular bounds signify the sole components of a test network

### Deep neural network

Our aim was to utilize deep learning to represent an RGB image as a VI, or in other words to estimate the VI from an RGB image.

#### Architecture

The architecture is a modification of the AlexNet deep convolutional neural network [34]. Deeper networks like ResNet [35] and GoogleNet [36] allow for more complex feature learning in diverse classes but also require much higher resolution input images to propagate through the net. Our choice of a DNN with a few hidden layers was suitable for content and resolution of input images.

The DNN maps a color image to a scalar VI as shown in Fig. 2. The training network is comprised of two convolution layers, two max-pooling layers, one mean-pooling layer, a dropout layer for regularization, and a fully connected layer. The input to the net is a three channel RGB color image of vegetation plot of $H \times W$ pixels. The input image passes through the first convolutional layer which extracts 20 feature maps with a $5 \times 5$ kernel. The resulting feature maps down-sample by max-pooling of non-overlapping $2 \times 2$ regions. The second convolutional layer extracts 50 feature maps with a $5$ kernel, followed by another max pooling operation of non-overlapping $2 \times 2$ pixels. The feature maps resulting from the second pooling layer connect to two inner product layers (fully connected) with an intermediate rectified linear unit layer and a dropout layer. The inner product layers successively reduce the dimensions of the feature maps down to a scalar. The output of the network is the activation of the (single neuron) final layer. The error was defined by a real-valued Euclidean loss function which computed the difference between the actual VI value and that estimated by the model.

**Fig. 3:**
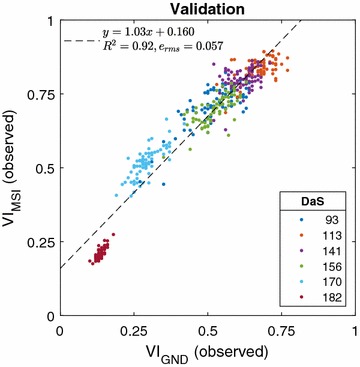
Comparison of ${{VI}}_{{MSI}}$ and ${{VI}}_{{GND}}$ of aerially observed $\text {VI}_{\text {MSI}}$ with ground reference ($\text {VI}_{\text {GND}}$) at different DaS for selected plot samples (Ground reference data for DaS 135 could not be recorded due to technical issue.). The equation, coefficient of determination ($\text {R}^2$) and Root Mean Squared Error ($\text {e}_{\text {rms}}$) of regression analysis is provided in legend

We used the *Caffe Deep Learning Framework* (BVLC, UC Berkeley) [37] for implementation of the design, training and validation of model. The experiments were performed on an Intel Xeon PC with 128GB RAM and a GeForce GTX TITAN X (NVIDIA, USA) graphics processing unit with CUDA enabled for faster computations.

**Fig. 4:**
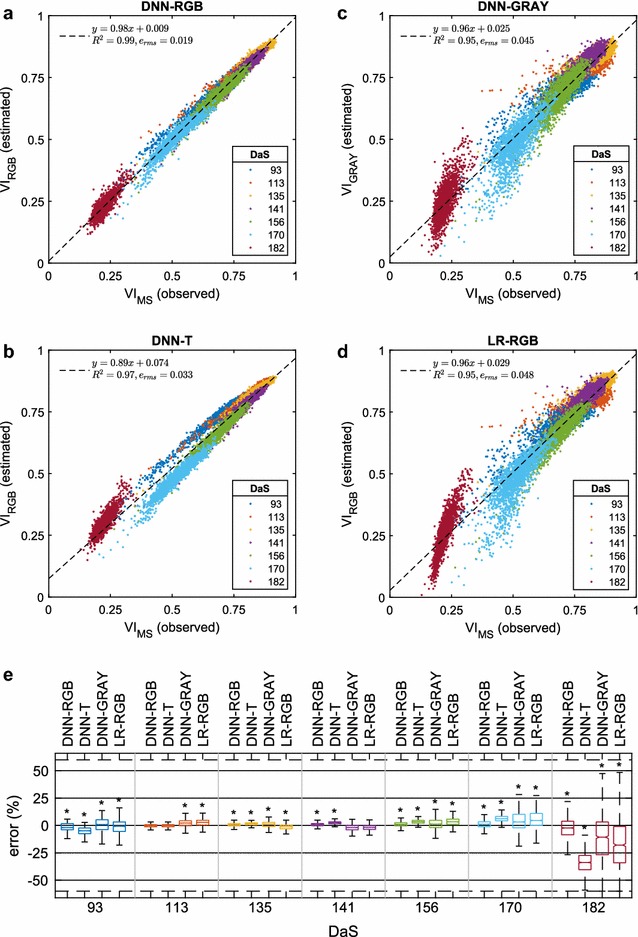
Comparison of the observed and estimated NDVI. **a** DNN-RGB, **b** DNN-T, **c** DNN-GRAY, **d** LR-RGB estimation model.The equation, coefficient of determination ($\text {R}^2$) and Root Mean Squared Error ($\text {e}_{\text {rms}}$) of regression analysis is provided in legend. **c** Error statistics of estimation models at different DaS. In each box, the central mark is median, and the lower and upper edges denote the 25th and 75th percentile of errors ($q_1$ and $q_3$), respectively. If the central notches of two boxes do not overlap, their true medians are different at the 0.05 significance level (indicated by *). The whiskers extend to the most extreme data points not considered outliers $[q_1-w\times (q_3 - q_1),q_3+w\times (q_3-q_1)], w=1.5$. Outliers not shown on the chart for clarity

#### Training

For training the network, RGB image data of vegetation plots was sampled from the multispectral image. The mean VI values were computed from the NIR and Red channels of the corresponding vegetation plots. Then, the network was trained with RGB images as the input source and the VI values as the target output. The Stochastic Gradient Descent algorithm was used to optimize the network weights by minimizing back-propagation error. The weights were iteratively updated so as to minimize the scalar distance (loss) between the output of the mean pooling layer and the final inner inner product layer. A mini-batch of 72 images was randomly sampled from the training set in each iteration. Moreover, the training data was augmented by randomly flipping the images. Training was conducted for the same number of epochs in each fold of validation. A fixed set of values for hyper-parameters was chosen for training across the folds. The base learning rate was initialized as $\alpha =0.01$ with a momentum $\gamma =0.9$ for quick convergence. The weight decay parameter was fixed as 0.0005. An inverse decay function defined the learning rate policy which reduced the learning rate with each iteration according to $\alpha \times (1+\gamma \times \text {iter})^{-\text {power}},$ where ${\mathrm{power}}=0.75.$

#### Testing

A test RGB image was forward propagated through the trained network to get the estimated index value from the final fully connected layer. Note that the dropout layer was excluded from the test network as its only purpose was to provide regularization for training.

## Results

All data was split into training and test sets for experiments. Robust Least Squares Regression was utilized to compare model accuracies. Root Mean Squared Error (RMSE) and coefficient of determination (R^2^) were used as the criterion for model evaluation. Pearson’s correlation coefficient (*r*) were also considered to assess the linear correlation between the observations and their estimates.

### Validation

We first validate the aerially observed VI by comparison with manually recorded ground measurements for 72 selected reference plots. Figure 3 provides a scatter plot of VI observed by UAV using multispectral imaging ($\text {VI}_{\text {MSI}}$) and VI measured by ground reference ($\text {VI}_{\text {GND}}$) at different growth stages (DaS). Note that the term growth stage here refers to imaging time points, and should not be confused with the phenological growth stage. It can be seen that both measurements are highly correlated (R^2^, RMSE = 0.057) across all growth stages.

**Fig. 5:**
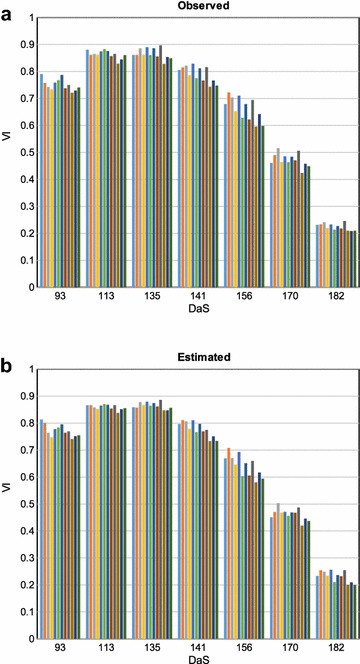
Intra-variety NDVI variation. **a** Observed NDVI values, and **b** estimated NDVI values for single row wheat plots of the Morocco variety across the growth stages (DaS). The twelve values at each growth stage represent independent replicates

It should be noted that the measurements are prone to methodological differences. The ground sensor’s field of view and region of interest chosen, result in measurements of different proportions of vegetation/soil regions. Moreover, the ground based sensor has an an active illumination source, whereas the aerial measurement utilizes solar illumination. A uniform lighting condition is assumed for the duration of the survey which may not always be valid on a particular day. Despite all differentiating factors, the validation model parameters suggest a significant correlation between the aerial and ground based measurements.

### Estimation of vegetation index

For index estimation, a DNN model was trained with RGB images of all growth stages. Models were trained in three fold cross validation, where in each fold, two spatially different bays were used for training and the held-out bay for testing. Therefore, the training set comprised 8064 samples (576 plots $$\times$$ 2 bays $$\times$$ 7 growth stages), whereas the test set constituted 4032 (576 plots $$\times$$ 1 bay $$\times$$ 7 growth stages) samples. We term it as DNN-RGB model which attempts to learn the relationship between RGB image and VI. The index observed from multispectral images ($$\text {VI}_{\text {MS}}$$) against the index estimated by a trained model using RGB image ($$\text {VI}_{\text {RGB}}$$) are presented in Fig. 4a. Regression analysis suggested that the RGB image estimated VI values had a good agreement with the observed VI values (R$$^2=0.99,$$$$e_{\text {rms}}=0.019$$). The contribution of spatial, spectral and temporal information of images to VI estimation can be validated as follows.

**Fig. 6:**
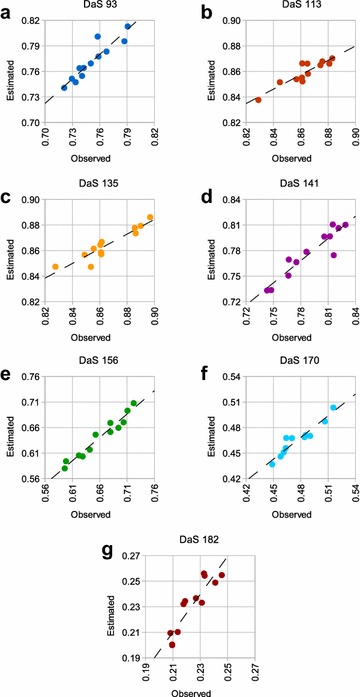
Analysis of variety across growth stages. **a**–**g** Relationships of observed and estimated NDVI for the Morocco variety across the growing season. The twelve values for each growth stage represent independent replicates.

#### Spectral information

The extent to which RGB color information contributed to vegetation index of a plot was quantified by the method of elimination. For this purpose, color information was removed from all RGB images by conversion to grayscale. Then a DNN was trained with the grayscale images as input and vegetation index as output. The trained model (DNN-GRAY) was utilized to estimate VI of plots given test grayscale images. The results were compared to that of a DNN trained on color images and the differential loss was examined to quantify the advantage of color information. The grayscale estimated index ($\text {VI}_{\text {GRAY}}$) is plotted against the multispectral observed index ($\text {VI}_{\text {MS}}$) in Fig. 4c. The root mean square error using grayscale image based VI estimation model was found to be more than twice ($e_{\text {rms}}=0.045$) in comparison to that of RGB image based model. It demonstrates that RGB does contribute useful information for estimation of VI.

#### Spatial information

The contribution of spatial information in images of vegetation to estimated VI was quantified by purging the spatial dimension. To achieve this objective, spatial information was reduced from all RGB images (by taking the average of pixels in each channel) to a single pixel ($1\times 1\times 3$) image. Then a linear regression model was learned with the spatially diminished images as predictor variable and vegetation index as the response variable. The trained regression model (LR-RGB) was used to estimate VI of test plots given single pixel images. The results were compared to that of DNN trained on original RGB images and the differential loss was evaluated to quantify the advantage of spatial information.

**Fig. 7:**
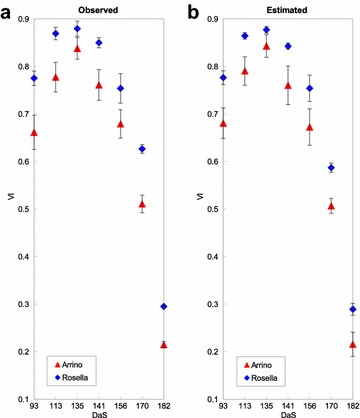
Inter-variety NDVI variation. **a** Observed NDVI values, and **b** estimated NDVI values across the growing season for two wheat varieties. The values are mean ± standard deviation of 4 replicate plots.

#### Temporal information

The effect of temporal information of VI at different growth stages on estimated vegetation index was evaluated. To this end, RGB images of one growth stage were withheld for training. In this manner, a learned DNN model was made temporally blind to images of one growth stage. For obtaining results, training was done on 10368 images (576 plots $\times$ 3 bays $\times$ 6 growth stages). The trained model (DNN-T) was tested on 1728 images of the left-out growth stage (576 plots $\times$ 3 bays $\times$ 1 growth stage). The process was repeated in a similar manner for all growth stages. It is encouraging to see that the DNN predicted VI at unseen growth stages by making use of information in nearby growth stages. It should be noted that a considerably different distribution of VI of the training data from the test data likely resulted in increased estimation error (e.g. DaS 182).

**Table 2:** Percentage estimation error statistics, mean (*μ*), standard deviation (*σ*), and the correlation coefficient (*r*) of the observed and estimated VI of each model

DaS	Model	$\mu \pm \sigma$	*r*
93	DNN-RGB	− 1.78 ± 4.03	0.97
	DNN-GRAY	0.00 ± 7.40	0.86
	DNN-T	− 5.34 ± 4.30	0.98
	LR-RGB	− 1.68 ± 6.92	0.89
113	DNN-RGB	− 0.61 ± 2.38	0.98
	DNN-GRAY	1.41 ± 5.67	0.68
	DNN-T	− 0.56 ± 2.32	0.99
	LR-RGB	1.91 ± 5.66	0.70
135	DNN-RGB	0.50 ± 1.73	0.96
	DNN-GRAY	0.70 ± 3.22	0.84
	DNN-T	1.27 ± 1.62	0.97
	LR-RGB	− 1.57 ± 2.90	0.89
141	DNN-RGB	0.87 ± 1.82	0.97
	DNN-GRAY	− 2.07 ± 3.14	0.90
	DNN-T	2.53 ± 1.74	0.97
	LR-RGB	− 1.97 ± 2.98	0.91
156	DNN-RGB	1.09 ± 2.51	0.96
	DNN-GRAY	2.08 ± 6.89	0.88
	DNN-T	3.46 ± 2.18	0.97
	LR-RGB	4.07 ± 5.91	0.93
170	DNN-RGB	1.45 ± 4.42	0.95
	DNN-GRAY	6.78 ±14.78	0.80
	DNN-T	6.62 ± 4.02	0.96
	LR-RGB	9.14 ±17.84	0.81
182	DNN-RGB	− 1.81 ± 9.56	0.83
	DNN-GRAY	− 8.13 ±23.21	0.73
	DNN-T	− 28.83 ± 7.13	0.86
	LR-RGB	− 11.46 ±26.03	0.87

Table 2 summarizes the error statistics and correlation at each growth stage for DNN-RGB, DNN-GRAY, DNN-T and LR-RGB models. The errors were calculated as a relative difference of the observed and estimated VI using,

1 $$\begin{aligned} \text {error } (\%) = \frac{\text {VI}_{\text {obs}}-\text {VI}_{\text {est}}}{\text {VI}_{\text {obs}}}\times 100 \end{aligned}$$

Figure 4e graphically illustrates the statistics of the errors. It can be observed that DNN-RGB consistently outperforms all other methods. The estimation errors were considerably larger for the DNN-T model compared to DNN-RGB model. This is not surprising since the DNN-T model does not recognize spatio-spectral variations at all temporal stages. Similarly, DNN-GRAY model is unable to sufficiently distinguish vegetation and background since it is not familiar with vegetation color resulting in unreliable VI estimates. In contrast, as the scope of a DNN-RGB model is complete, so it is familiar with all the spatial, spectral and temporal variations in VI. In future work, a more complex DNN model could be designed to account for causal relationships of the data by using recurrent neural networks [38].

A significantly higher error was observed by all methods upon senescence (DaS 170, 182). It showed difficulty in accurately modeling relationship of mature plant RGB images and their unique range of VI. Larger errors for LR-RGB model suggested a highly non-linear relationship of RGB images and VI after maturity. It also explained the likely reason for the failure of DNN-T model in mature growth stage, since it was blind to images of that stage. The DNN-T model estimates had high correlation with observed VI, albeit the estimates were biased and resulted in higher average error. In contrast, the DNN-RGB model demonstrated relatively lower errors and its average error consistently remained within ± 2% at all growth stages.

A multispectral camera was used for the study to directly compare the RGB image estimated NDVI with a multispectral image observed NDVI. Thus, training was performed on low resolution RGB images sourced from the multispectral sensor. However, the proposed methodology can be extended to high-resolution RGB cameras. A common approach to adapt to a DNN where the input image size differs from the network input is to resize the input image. Therefore, DNN models trained on low resolution RGB images can be extended to an RGB camera by resizing the images.

#### Phenotyping with VI estimation

In order to evaluate the utility of the proposed approach for phenotyping in breeding experiments, we observed if the intra-variety and inter-variety differences were preserved in VI estimation. For this purpose, we selected the Morocco variety as a check-line in the trials and had 12 replicates allowing comparisons throughout the season. As shown in Fig. 5, the variation in estimated NDVI across the season was consistent with the observed NDVI. Specifically, the trend of observed NDVI within replicates at each growth stage was largely preserved in estimated NDVI values as well.

Using Morocco again as an example, the relationship between the observed and estimated NDVI values was much clearer when plotted individually at each growth stage as shown in Fig. 6. Despite the large changes in NDVI across the season, and the relatively small variability in NDVI of replicates at each growth stage, there was a strong relationship between the observed and estimated values.

In terms of comparison of observed and estimated NDVI across varieties, we selected Arrino and Rosella variety, each with 4 replicates. Relatively subtle differences in observed NDVI between the varieties were also preserved in the estimated NDVI values as shown in Fig. 7. Moreover, the VI variability within a variety was found to be preserved relative to the variability between varieties.

An important consideration is the fact that no genotype-specific information was considered in learning of the estimation models. This helps show the robustness of the modeling approach as genotypic variation in growth characteristics would be a source of error in the observed and estimated NDVI relationships.

## Conclusion

The use of RGB cameras and UAVs provide a ubiquitous solution for calculating VI for high throughput precision agriculture. This comes at a cost of an estimate of VI rather than actual VI. However, as demonstrated by our experiments, the tradeoff minimally affects the reliability of measurement. The current study was based on single row wheat plants and further analysis will be required to evaluate the feasibility of the proposed approach in broad acre crops. This could include estimation of the VI image of a paddock (instead of the VI of a plot) using an RGB image. In addition, the generalization of this approach by application to other crops of interest will be of significant value.
